# Positive-unlabeled learning for the prediction of conformational B-cell epitopes

**DOI:** 10.1186/1471-2105-16-S18-S12

**Published:** 2015-12-09

**Authors:** Jing Ren, Qian Liu, John Ellis, Jinyan Li

**Affiliations:** 1 Advanced Analytics Institute, Faculty of Engineering and Information Technology, University of Technology Sydney, Sydney, NSW 2007, Australia; 2 Advanced Analytics Institute, Faculty of Engineering and Information Technology, University of Technology Sydney, Sydney, NSW 2007, Australia; 3 School of Life Sciences, University of Technology Sydney, Sydney, NSW 2007, Australia; 4 Advanced Analytics Institute and Centre for Health Technologies, University of Technology Sydney, Sydney, NSW 2007, Australia

**Keywords:** epitope prediction, positive-unlabeled learning, unbound structure, epitopes of Ebola antigen, species-specific analysis

## Abstract

**Background:**

The incomplete ground truth of training data of B-cell epitopes is a demanding issue in computational epitope prediction. The challenge is that only a small fraction of the surface residues of an antigen are confirmed as antigenic residues (positive training data); the remaining residues are unlabeled. As some of these uncertain residues can possibly be grouped to form novel but currently unknown epitopes, it is misguided to unanimously classify all the unlabeled residues as negative training data following the traditional supervised learning scheme.

**Results:**

We propose a positive-unlabeled learning algorithm to address this problem. The key idea is to distinguish between epitope-likely residues and reliable negative residues in unlabeled data. The method has two steps: (1) identify reliable negative residues using a weighted SVM with a high recall; and (2) construct a classification model on the positive residues and the reliable negative residues. Complex-based 10-fold cross-validation was conducted to show that this method outperforms those commonly used predictors DiscoTope 2.0, ElliPro and SEPPA 2.0 in every aspect. We conducted four case studies, in which the approach was tested on antigens of West Nile virus, dihydrofolate reductase, beta-lactamase, and two Ebola antigens whose epitopes are currently unknown. All the results were assessed on a newly-established data set of antigen structures not bound by antibodies, instead of on antibody-bound antigen structures. These bound structures may contain unfair binding information such as bound-state B-factors and protrusion index which could exaggerate the epitope prediction performance. Source codes are available on request.

## Background

A B-cell epitope is a small surface area of an antigen that interacts with an antibody. It is a much safer and more economical target than an entire inactivated antigen for the design and development of vaccines against infectious diseases [1,2]. More than 90% of epitopes are conformational epitopes which are discontinuous in sequence but are compact in 3D structure after folding [2,3]. The most accurate way to identify conformational epitopes is to conduct wet-lab experiments to obtain the bound structures of antigen-antibody complexes. Given that there are a vast number of antigen and epitope candidates for known antigens, the wet-lab approach is unscalable and labour-intensive.

The computational approach to identify B-cell epitopes is to make predictions for new epitopes by sophisticated algorithms based on the wet-lab confirmed epitope data. Early methods explored the use of essential characteristics of epitopes, and found useful individual features including hydrophobicity [4,5], flexibility [6], secondary structure [7], protrusion index (PI) [8], accessible surface area (ASA), relative accessible surface area (RSA) and B-factor [9,10]. However, none of these single characteristics is sufficient to locate B-cell epitopes accurately. Later, advanced conformational epitope prediction methods emerged, integrating window strategies, statistical ideas and compound features [2,11-14]. Recently, many epitope predictors have used machine learning techniques, such as Naive Bayesian learning [15] and random forest classification [10,16].

All these methods have overlooked the incomplete ground truth of the training data of epitopes. The training data is simply divided into positive (i.e., confirmed epitope residues) and negative (i.e., non-epitope residues) classes by the traditional methods. In fact, the non-epitope residues are unlabeled residues. These unlabeled residues may contain a significant number of undiscovered antigenic residues (i.e., potentially positive). It is therefore misguided to unanimously treat all the unlabeled residues as negative training data. Classification models based on such biased training data would significantly impair their prediction performance.

An intuitive way to address this problem is to train the models on positive samples only (one-class learning). One-class SVM [17,18] was developed, but its performance does not seem to be satisfactory [19]. Positive-unlabeled learning (PU learning) provides another direction. It learns from both positive and unlabeled samples, and exploits the distribution of the unlabeled data to reduce the error labels of training samples to enhance prediction performance [19]. One idea in PU learning is to assign each sample a score indicating the probability of it being a positive sample. For example, Lee and Liu first fitted samples with specific distribution by weighted logistic regression and then scored the samples [20]. Another idea is the bagging strategy, in which a series of classifiers is constructed by randomly sampling unlabeled data, and these classifiers are then combined using aggregation techniques [21]. A third idea is a two-step model: reliable negative (RN) samples from unlabeled data are first obtained, then a classifier is built by applying a classification algorithm on the positive and reliable negative samples [19,22-24].

We introduce a novel two-step PU learning algorithm. The first step is to identify reliable negative samples from unlabeled data by a weighted SVM [25] with a recall threshold set at a high level. The high recall means that the majority of positive samples should be correctly identified; thus if an unlabeled sample is predicted as negative, it would have a high probability of being a non-epitope residue. Accordingly, true negative predictions (i.e., unlabeled residues predicted as negative) can be annotated as reliable negative samples. A classifier (a weighted SVM model) is then trained on the positive and reliable negative samples to predict novel antigenic resides and epitopes. Our method is called PUPre (*P*ositive- *U*nlabeled *Pre*diction).

The performance of PUPre was evaluated on a newly-established data set of *unbound structures *of antigens. We would like to point out that most existing epitope prediction methods have been evaluated on bound-state structures of antigens [2,11,13,26]. Bound-state data has two limitations. Firstly, bound-state structures contain binding site information [10]; Secondly, if an antigen can be bound by multiple antibodies, only one epitope is annotated as an epitope in a bound-state structure, while those epitopes bound to the other antibodies are taken as non-epitope. Such an annotation exaggerates the false negative annotations.

We conducted complex-based 10-fold cross-validation for performance evaluation, in which all the residues of 10% randomly selected complexes are reserved for test at each round (not 10% of randomly selected residues). We show that the PUPre method demonstrates better performance compared to commonly used conformational B-cell epitope predictors, such as DiscoTope 2.0, ElliPro and SEPPA 2.0. The use of PUPre was also demonstrated through its application to antigens of West Nile virus, dihydrofolate reductase, beta-lactamase, and two Ebola antigens (whose epitopes are currently unknown) to show its usefulness in real-life applications for the prediction of unknown epitopes. To understand the importance of species information in epitope prediction, a species-wise feature analysis was also conducted on the newly-established unbound structure data set. We found that the divergence between epitopes and normal surface areas is large, suggesting that the prediction methods are useful for all species.We note that a difference exists between certain species on important structural features and amino acid composition. We speculate that it may be possible to enhance epitope prediction performance by using species information in the future.

## Methods

### Data sets

Large-scale bound-state structure data sets have previously been constructed by the literature, and used in other studies [2,11,13,26,27] for epitope prediction and feature analysis. The use of bound-state structures can result in two problems. One is that bound-state structures contain a large amount of explicit binding information [10], which can result in biased characterization of epitopes and can exaggerate the prediction performance. The other is that they can aggravate the issue of false negative annotations when an antigen can be bound by multiple antibodies--only the epitope to the antibody in the bound structure is labeled as an epitope site and all those epitopes to other antibodies are marked as non-epitope. To overcome these two problems, our predictions and analysis were based on a newly-established unbound-state structure data set. As the data set does not contain information about the binding site, a more accurate characterization of unboundstate epitopes is expected. The use of unbound-state structures can also reduce the false negative annotations by aggregating multiple epitopes on the same antigen. These unbound-state structures were manually organized in terms of species and disease which is especially useful for species/disease-specific feature analysis.

The construction of the unbound-state structure data set requires reference information from bound structures. We used the following steps to obtain the bound structures with epitope annotations:

• Collect bound structures of antigen-antibody complexes. Bound structures were collected by text search of 'complex' and 'antibody/Fab/ Fv/VHH' from the PDB database dated 9th Sep 2014, which retrieved 1596 structures.

• Filter the bound structures. A bound structure was removed if it was consistent with any of the following conditions: (1) there is no antibody chain; (2) there is a chain of 'DNA/RNA/Fc/T-cell/receptor'; (3) the resolution is worse (more) than 3Å; (4) the antigen chain is less than 25 residues [2,28]. In total, 598 bound structures of antigen-antibody complexes were retained.

• Determine the location of epitopes: A residue of an antigen is considered to be an epitope residue if a heavy atom of the residue is within 4Å distance from any heavy atom of the antibody [2,13].

Subsequently, the steps to build the unbound-state structure data set are:

• Obtain candidate unbound-state structures of antigens. An antigen structure in unbound state is selected as a candidate if it has more than 70% sequence similarity to any antigen in bound state (i.e., the 598 bound structures). By this way, there may be multiple candidate unbound-state antigen structures that are similar to the same bound-state antigen, but only one will be used for mapping in the next step. The candidate with the highest similarity to the bound-state antigen and with higher resolution is considered to have higher priority. Bound-state structures will be removed if their antigens do not have high similarity to antigens in unbound state.

• Map the epitopes onto the unbound-state structures. The epitopes extracted from the bound structures were mapped onto the corresponding unbound-state structures by structure alignment. An epitope was retained if it could be completely aligned with the unbound structure. This step reduces the false negative annotations: if various bound structures share the same antigen, their epitopes will be mapped on the same unbound-state structure. For example, 1VFB and 2EIZ are bound structures of lysozyme and antibodies, and their distinct epitopes were mapped onto the same unbound-state structure 2VB1 to reduce the false negative annotations. In this step, 308 epitopes were mapped onto 92 unbound-state structures.

• Remove duplicate units. For the 92 unbound-state structures, only one asymmetric unit was retained for each structure.

A residue was retained for the unbound data set if its ASA was more than 0Å. This is because the candidate epitope residues at least need to be exposed to contribute to the binding affinity. We used the relative low threshold of 0Å to preserve the ground truth of the epitopes.

By following these steps, a data set of 92 unbound-state structures was constructed (Additional File 1) which contained 2123 confirmed epitope residues labeled as positive (Additional File 2), and 16615 residues marked as unlabeled.

### Feature vector representation for residues

Various features of amino acids were used together as a vector to represent a residue. We collected 239 basic features (Table 1), including 205 physico-chemical features collected from AAIndex with less than 80% similarity, 21 evolutionary features (PSSM features), and 13 structural features.

**Table 1:** Description of features.

Features	Collected from	**Feature No**.
Physico-chemical	AAIndex (80% similarity)	1-205
PSSM	Psi-BLAST	206-225
PSSM residue	PSSM	226
PI	PSAIA	227
Secondary structure	DSSP	228-235
ASA	NACCESS	236
RSA	NACCESS	237
B-factor	PDB files	238
B-factor CA	PDB files	239

'Feature No.' describes the number of dimensions for each feature type.

Some of these features may make little contribution to the characterization and identification of epitopes. A non-parameter hypothesis test (Wilcoxon rank-sum test on the epitope residues and the unlabeled residues) was used to find out which features better characterized epitopes. The p-value reflects the significance: the smaller the p-value is, the better the feature characterizes epitope residues. The features are then ranked by p-values. Only those with p-value less than 1*e - *4 were retained as important features. To avoid over-fitting, the hypothesis test was conducted on 62 (2/3 of 92) randomly selected complexes each time. This procedure was repeated nine times, and produced nine important feature lists. The final winning features were selected by majority voting. The procedure helps to identify highly useful features for discovering unknown epitopes. Eighty-nine basic features were ultimately selected. Sequence window features and structure window features were also added to the vector to reflect the impact of sequential or structural adjacent residues on epitope residues. Please refer to [10] for detailed steps to derive these sequence window features and structure window features.

### PU learning

PU learning has been already explored for text mining [19,20,22], disease gene identification [29-31], and protein function identification [32]. However, this advanced learning approach has not been explored for the prediction of epitopes.

As mentioned, some conventional PU learning algorithms share a two-step framework. The difference between them lies in the unique strategies used in the first or the second step. Table 2 summarizes the differences. The spy strategy [19] randomly selects several positive samples as spies and puts them into the unlabeled data set; then it determines the boundary of RN (reliable negative samples) under the rule that most of the spies are classified as positive. The 1-DNF algorithm [22] identifies the reliable positive features, and then selects RN which consist of none of the reliable positive features. Rocchio [22] calculates the representative positive/unlabeled vectors and selects those samples more similar to the representative unlabeled vector as RN.

**Table 2:** Typical two-step PU learning algorithms.

Algorithms	Step1	Step2	SEL
S-EM [19]	Spy	EM	Y
PEBL [22]	1-DNF	SVM	N
Roc-SVM [23]	Rocchio	SVM	Y
Biased SVM [24]	Naïve Bayesian	Biased SVM	N

SEL: whether to select the final model from the iterated classifiers.

**Figure 1:**
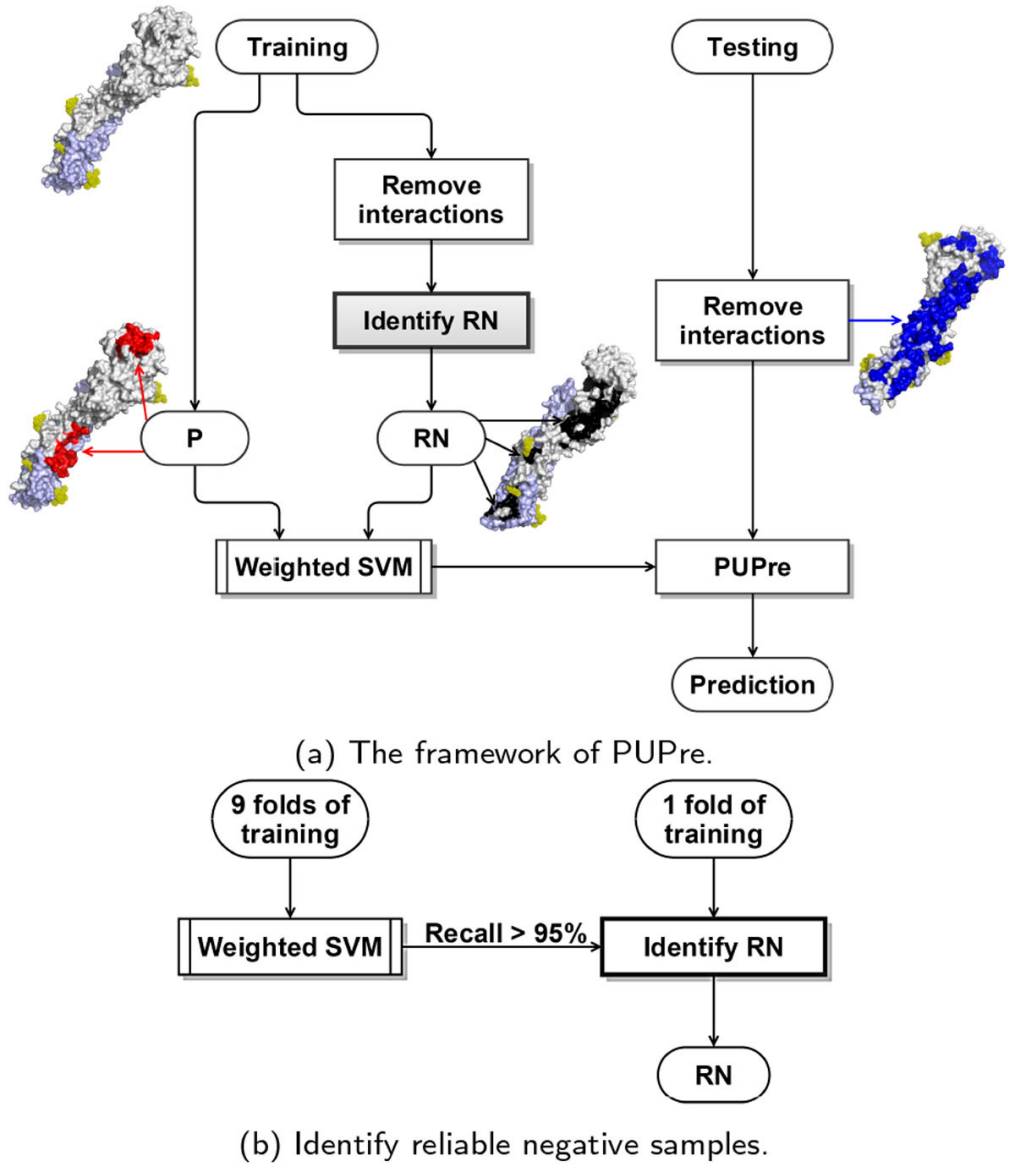
**The learning process of PUPre**. P is short for positive samples (i.e., epitope residues), and RN is an acronym for reliable negative samples. (b) is a detailed description of the step 'Identify RN' in (a).

We introduce a novel two-step PU learning algorithm (i.e., PUPre) based on weighted SVM with linear kernel [25]. For easy reference, we list three groups of terms and notations which can be used interchangeably: (1) P, positive samples and epitope residues, (2) U, unlabeled samples and unlabeled residues, (3) RN, reliable negative samples and reliable non-epitope residues. Figure 1(a) shows the two main steps of PUPre: identify RN and construct a weighted SVM from P and RN. Figure 1(b) details the step 'identify RN', where a high recall (e.g., *>*95%) for the weighted SVM is highlighted. The high recall requires that the majority of epitope residues should be correctly identified. Thus if an unlabeled residue is predicted as negative, it would have a high probability of being a nonepitope residue. Note that although the two steps both use weighted SVM, their optimization objectives are slightly different. In the first step, the true negative predictions (i.e., unlabeled residues predicted as negative) are selected as RN, with the optimization objective that the recall of the predictor is as high as 95% and the F-score is optimized. The 5% balance in recall is reserved to tolerate noise in positive samples induced by computational definitions and the mapping process. In the second step, the PUPre model is developed with the objective of optimizing the F-score. The function of weighted SVM can vary when the optimization function function is adjusted. In addition, adjusting the weights assigned to different classes can help to deal with the issue of data imbalance between positive residues and a huge amount of unlabeled residues. Both the parameter penalty factor and weight used in the two steps were selected by maximizing the optimization goals. The parameter determination process was conducted under internal complex-based 5-fold cross-validation to avoid over-fitting.

To study which factor contributes to this major improvement, we also designed two baseline algorithms using linear SVM. In the baseline algorithms, we simply use weight = 10 on the rare epitope data to handle the issue of data imbalance, and calibrate the penalty factor *cc *in linear SVM to obtain optimum performance. The penalty factor controls the trade-off between the margin and the training errors [25].

The raw baseline algorithm is trained and evaluated on all the epitope and unlabeled residues. It is used to investigate whether selected features are effective. The preprocessing baseline algorithm was designed on the basis of the following observation. Many of the unbound structures are multimeric, and in most cases PDB files only record parts of the symmetric units. Clearly, the interfaces between target chains and other chains in symmetric units cannot become epitopes. Thus, a preprocessing procedure is deployed to enhance the performance: we first calculate the complete structure according to the PDB file and then detect and remove the interfaces with other chains. Without loss of generality, we assume an antigen structure has two chains A and B. A residue on chain A is defined as an internally interacting residue if a heavy atom of this residue is within 4Å distance of any heavy atom of chain B. In a training process, those internal interactions are excluded from the training data, taken as neither positive nor unlabeled residues; in a testing process, they are labeled as negative. The internal interactions on our data set are provided in Additional File 3.

**Figure 2:**
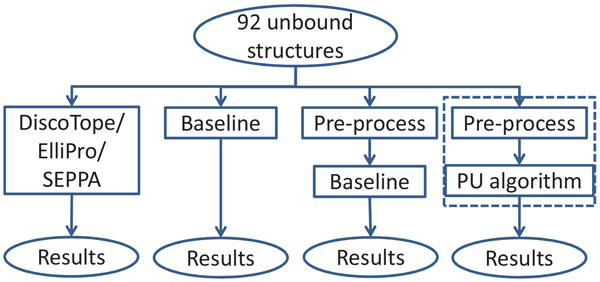
**Performance evaluation and comparison**. The proportion inside the dotted box is referred to as PUPre.

In the performance comparison and evaluation, complex-based 10-fold cross-validation was used. By this process, the 92 unbound structures were randomly divided into 10 groups. Our PUPre model was trained on the complexes from nine groups, and tested on the remaining group. Complex-based 10-fold cross-validation is an excellent indicator of performance in real world applications, because when predictors make predictions on an antigen structure, the complete antigen should be taken as testing data, and none of the residues should be in the training data. However, residue-based cross-validation is quite likely to divide residues from the same antigen (or even nearby epitope residues) into training and testing data, overstating the performance. Thus, it cannot reflect the true prediction performance on a new structure. Figure 2 demonstrates the evaluation of PUPre and the comparison with other predictors.

## Results and Discussion

This section presents validation results in two parts. The performance of PUPre under the complex-based 10-fold cross-validation is reported first, followed by its detailed prediction performance on the four antigens in our case studies. Our methods and all the comparing partners in this section receive exactly the same inputs: all residues of the chains listed in Additional File 1.

### Prediction results by complex-based 10-fold cross-validation

Complex-based 10-fold cross-validation was conducted on the unbound structure data set with random partition of the 92 complexes (Additional File 1). The random partitions were conducted three times to reduce the possible bias caused by the partition process. The mean and standard deviation of the performance are reported in Table 3. When compared with the three structure-based epitope predictors DiscoTope 2.0, ElliPro and SEPPA 2.0, it is clear that the PUPre classifier outperforms their prediction results in every aspect. In particular, PUPre achieves an F-score of 0.28, while the best F-score of the three predictors is 0.24 (by SEPPA 2.0). The MCC of PUPre is 0.21, which is 50% higher than the best MCC of the comparison predictors.

**Table 3:** The performance of complex-based 10-fold cross-validation.

Predictor	Recall	Precision	F-score	MCC
DiscoTope 2.0	0.26	0.17	0.21	0.11
ElliPro	0.68	0.12	0.20	0.08
SEPPA 2.0	0.48	0.16	0.24	0.14
Baseline(r)	0.58 ± 0.002	0.17 ± 0.003	0.26 ± 0.003	0.17 ± 0.004
Baseline(p)	0.59 ± 0.001	**0.18 ± 0.003**	0.27 ± 0.003	0.18 ± 0.004
**PUPre**	**0.71 ± 0.015**	**0.18 ± 0.002**	**0.28 ± 0.003**	**0.21 ± 0.005**

Baseline(r) stands for baseline(raw), baseline(p) is baseline(preprocessing).

The most distinguishing feature of PUPre is its high recall performance. It achieves an excellent recall of 0.71 while its precision is the highest level 0.18 of the four predictors. This indicates that most of the epitope residues have been correctly identified. Though ElliPro has a competitive recall, its precision of 0.12 is only slightly better than random $\left(\frac{2123}{2123+16615}\approx 0.113\right)$.

We can also see that the raw baseline algorithm (Table 3, Baseline(r)) outperforms the three other predictors except that its recall is lower than ElliPro. This implies that the selected features and the method of feature space construction are as effective as expected. By integrating the preprocessing procedure, the new baseline algorithm improves performance in every aspect: the F-score has increased from 0.26 to 0.27, the MCC has improved from 0.17 to 0.18, the recall has increased from 0.58 to 0.59 (implying that more epitope residues have been identified), and precision has increased from 0.17 to 0.18 (implying that a greater proportion of predicted epitope residues are true epitope residues). The removal of internally interacting residues was conducted as a preprocessing step rather than postprocessing. Thus, these extreme negative cases can be removed before training, and it will help predictors to focus on the more confusing residues. The performance results of DiscoTope 2.0, ElliPro, SEPPA 2.0 with a similar postprocessing procedure to remove the internally interacting residues are shown in Additional File 4: Table S1.

Compared with the two baseline algorithms, PUPre achieves an overall improvement in performance: the F-score has increased from 0.27 to 0.28 and the MCC has improved from 0.18 to 0.21. With precision unchanged, recall shows a significant increase from 0.59 to 0.71, indicating the effectiveness of the PU learning algorithm: more confirmed epitope residues are re-discovered (predicted) and there is potential to discover new epitopes.

In epitope prediction, handling the more ambiguous residues has always been difficult. The nature of epitope residues is complicated; simply using the distribution of certain features (even important features) is insufficient to distinguish these 'middle points'. Additional File 5: Figure S1 gives an example to illustrate this difficulty. As can be seen, there is no clear boundary among these samples that is able to correctly classify the determined epitope residues (positive). A more systematic machine learning method could be a better choice, to utilize the distribution of more useful features. In PUPre, two strategies were employed against these ambiguous residues. The first strategy was the preprocessing procedure to remove positive samples before step one. These internally interacting residues are a kind of extremely negative residue, and were removed before training and testing. Thus, the predictor is able to focus on the more ambiguous points. The second strategy was to train a new SVM predictor with optimized F-score based on the positive and RN residues. The distribution of positive and RN residues was utilized in this more systematic way to distinguish the more ambiguous points that were not labeled in step one.

### Four case studies

PUPre was tested on three antigens with known epitopes which were not used as training data to see whether these known epitopes can be correctly re-discovered (predicted). PUPre was also applied to two Ebola virus antigen structures, whose epitopes are currently unknown, to predict novel epitopes. We note that all these antigens have a far kinship from any antigen in the training data.

#### Prediction results for an antigen of West Nile virus

PDB entry 4OIE is an unbound structure of an antigen of West Nile virus. The epitope site of 4OIE was annotated with the reference information from PDB entry 4OII (protein NS1 of the West Nile virus binding with antibody 22NS1), which is the only bound structure of a similar antigen in PDB. The epitope consists of 21 residues. PUPre was constructed on 91 structures of our data set to predict the epitopes of 4OIE after the unbound structure 4OIE was removed from the training data. The sequence similarity between 4OIE and each of the 91 training structures was calculated by BLAST; the highest sequence similarity is only at 11.0%, confirming they are not related.

The prediction performance is listed in Table 4. We can see that PUPre outperforms the other three predictors in all cases. It has an F-score of 0.52 and an MCC of 0.49, both of which are significantly higher than the others. Its recall is high at 0.90, nearly twice as much as the best recall of the other predictors (ElliPro: 0.48). Its precision is also remarkably higher than all the others. The 0.90 recall means that 90% of the confirmed epitope residues have been correctly identified, and thus it can be inferred that most of the unknown epitope sites will probably also be predicted as positive. A high precision means that the number of candidate epitope residues is greatly reduced to ease the wet-lab burden of experiments.

**Table 4:** Prediction results on West Nile virus 4OIE.

Predictor	Recall	Precision	F-score	MCC
**PUPre**	**0.90**	**0.37**	**0.52**	**0.49**
DiscoTope 2.0	0.24	0.20	0.22	0.10
ElliPro	0.48	0.11	0.17	-0.05
SEPPA 2.0	0.14	0.14	0.14	0.02

**Figure 3:**
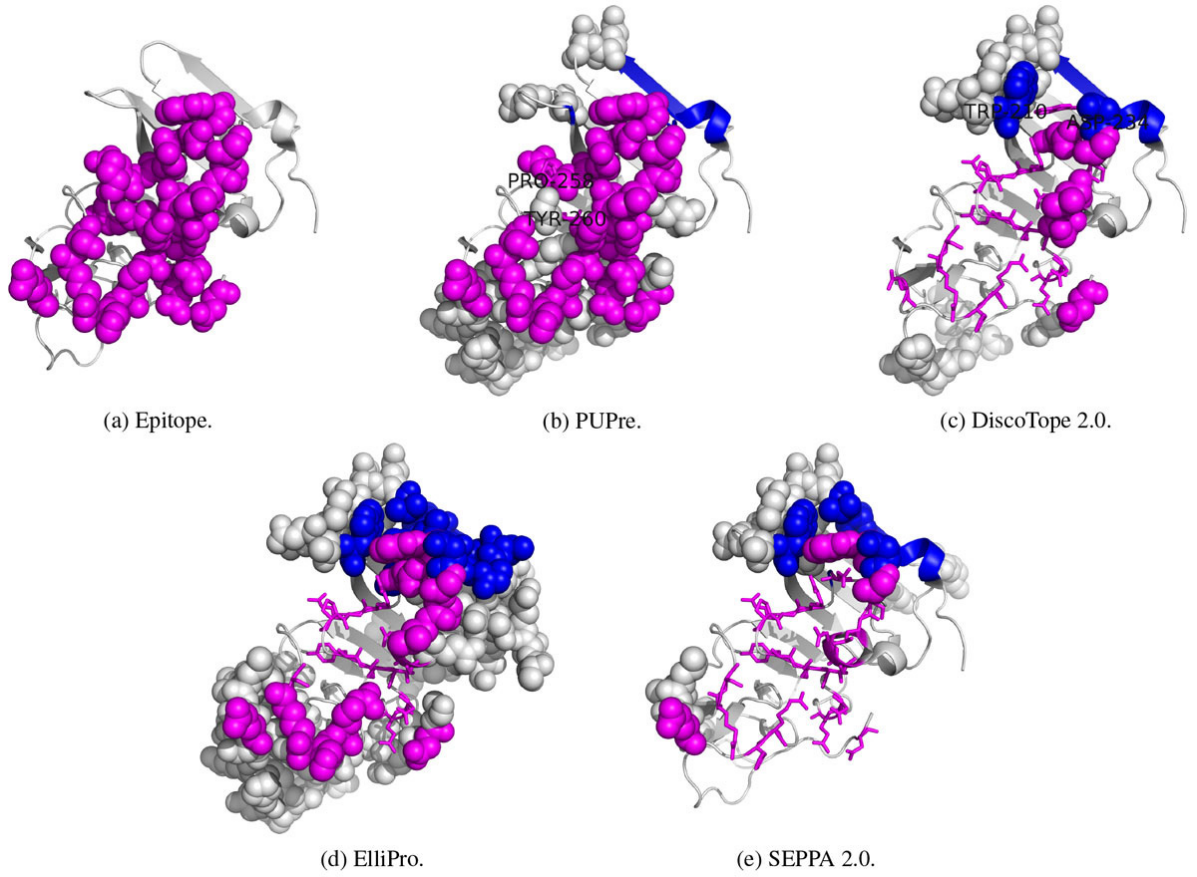
**Epitope prediction on a West Nile virus unbound structure (PDB ID: 4OIE)**. Magenta denotes the confirmed epitope sites, and blue denotes the internal interactions by other chains; predicted epitopes by each predictor are highlighted by spheres, while the epitope sites that fail to be identified (false negative) are shown as sticks.

Figure 3 shows the 21 confirmed epitope residues in comparison with the predicted epitope results from the four computational methods. The confirmed epitope (ground truth, may be incomplete) is shown in Figure 3(a) by the magenta spheres. The PUPre classifier made a correct prediction for 19 of the 21 epitope residues as highlighted by magenta spheres in Figure 3(b), except for PRO-258 and TYR-260 of chain A (shown by magenta sticks and labels). In addition, a total of 33 unlabeled residues were predicted as positive (shown as grey spheres). These residues are believed to be good candidates for currently unknown epitopes. As a close comparison, ElliPro made a correct prediction for only 10 of the 21 epitope residues (magenta spheres in Figure 3(d)), and it selected 84 unlabeled residues as potential epitope residues. DiscoTope and SEPPA's performance were worse than ElliPro's.

There are multiple symmetrical units in 4OIE. The footprint of other symmetrical units on this chain (internal interactions, colored in blue) cannot be epitope candidates. The integrated preprocess allows PUPre to avoid recognizing this area as an epitope site (there is no blue sphere in Figure 3(b)), while the other predictors all mistook internally interacting residues for epitope sites. For example, DiscoTope 2.0 predicted two internally interacting residues TRP-210 and ASP-234 as epitope residues (see blue spheres in Figure 3(c)).

#### Prediction results for dihydrofolate reductase antigen

PDB entry 4NX7 is an unbound structure of an antigen of dihydrofolate reductase. This structure has multiple epitopes that have been confirmed at six other PDB entries: 3K74, 4EJ1, 4EIG, 4EIZ, 4I1N and 4I13. Again, the PUPre classifier was trained on the remaining 91 unbound structures after 4NX7 was removed from the unbound structure data set. The best sequence similarity between 4NX7 and the remaining 91 structures is only 12.6%.

Table 5 reports that PUPre achieves the best overall performance: the F-score is 0.49 and the MCC is 0.31, noticeably higher than the other prediction methods. It recognizes 71% of the confirmed epitope residues at a precision of 0.37. ElliPro shows a competitive recall of 0.69, but its precision 0.29 is worse than ours; SEPPA 2.0 shows a slightly higher precision performance (0.38), but its recall is quite low (0.17).

**Table 5:** Prediction results on dihydrofolate reductase 4NX7.

Predictor	Recall	Precision	F-score	MCC
**PUPre**	**0.71**	0.37	**0.49**	**0.31**
DiscoTope 2.0	0.11	0.29	0.16	0.05
ElliPro	0.69	0.29	0.40	0.17
SEPPA 2.0	0.17	**0.38**	0.24	0.13

**Figure 4:**
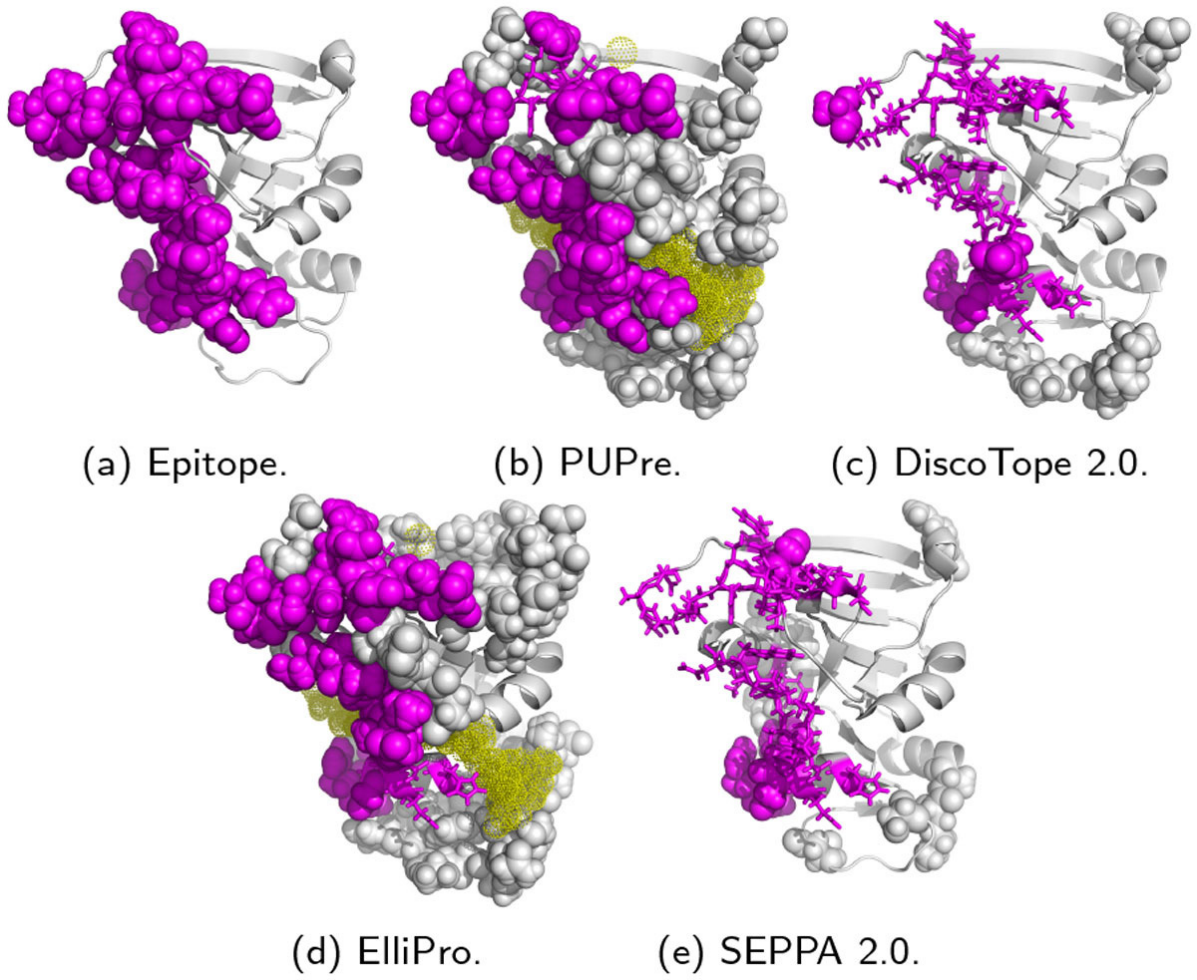
**Epitope prediction on a dihydrofolate reductase unbound structure (PDB ID: 4NX7)**. Magenta denotes the confirmed epitope sites; predicted epitopes by each predictor are highlighted by spheres, while the epitope sites that fail to be identified (false negative) are shown as sticks. The yellow dots are not standard amino acids, but are essential for the folding or the function of the protein.

A graphical visualization of the ground truth of the epitopes (total 35 residues) is given in Figure 4(a). Figure 4(b) shows the 25 correctly predicted residues by PUPre (magenta spheres) of the 35 epitope residues, and the 10 wrongly predicted epitope residues (magenta sticks). As a comparison, Figure 4(d) shows the 24 epitope residues correctly predicted by ElliPro, but ElliPro selected many more unlabeled residues (60, grey spheres) as being positive than PUPre (43 residues), thereby greatly reducing precision. SEPPA demonstrated a slightly higher precision than PUPre as it predicted only 10 unlabeled residues as epitope sites; however, it identified only six out of 35 epitope residues, implying limited ability to discover epitope sites compared to PUPre (Figure 4(e)).

We also use this case study to illustrate the impact of non-standard components on epitope prediction. There are four non-standard components in 4NX7 (in yellow dots in Figure 4(b) and (d)): MN (Manganese), FOL (Folic acid), BME (Betamercaptoethanol) and NAP (NADP nicotinamide-adenine-dinucleotipe). The nonstandard elements have a sophisticated impact on the folding of the protein as well as the binding with antibodies. As shown in Figure 4(b) and (d), the residues alongside the non-standard elements are unlikely to be epitope candidates since they are difficult to bind by antibodies. Predictors and most feature extraction methods have failed to deal with this issue.

#### Prediction results for beta-lactamase antigen

Our third case study is on an unbound structure of an antigen of beta-lactamase obtained from *Bacillus licheniformis *(PDB ID: 2WK0). The epitope site was mapped from the bound structure 4M3K. There are two symmetrical Chains A and B in 2WK0, and Chain A is used here as an example. The best sequence similarity of 2WK0 with the training data (91 unbound structures) is at 16.7%.

Overall, PUPre performs significantly better than all the other predictors (Table 6). The F-score and MCC are 0.41 and 0.38 respectively. Although ElliPro successfully identifies all the epitope residues (i.e., a recall of 1.00), its precision is very low at 0.14, only half that of PUPre.

**Table 6:** Prediction results on beta-lactamase 2WK0.

Predictor	Recall	Precision	F-score	MCC
**PUPre**	0.75	**0.28**	**0.41**	**0.38**
DiscoTope 2.0	0.25	0.19	0.22	0.14
ElliPro	**1.00**	0.14	0.25	0.26
SEPPA 2.0	0.10	0.06	0.08	-0.03

**Figure 5:**
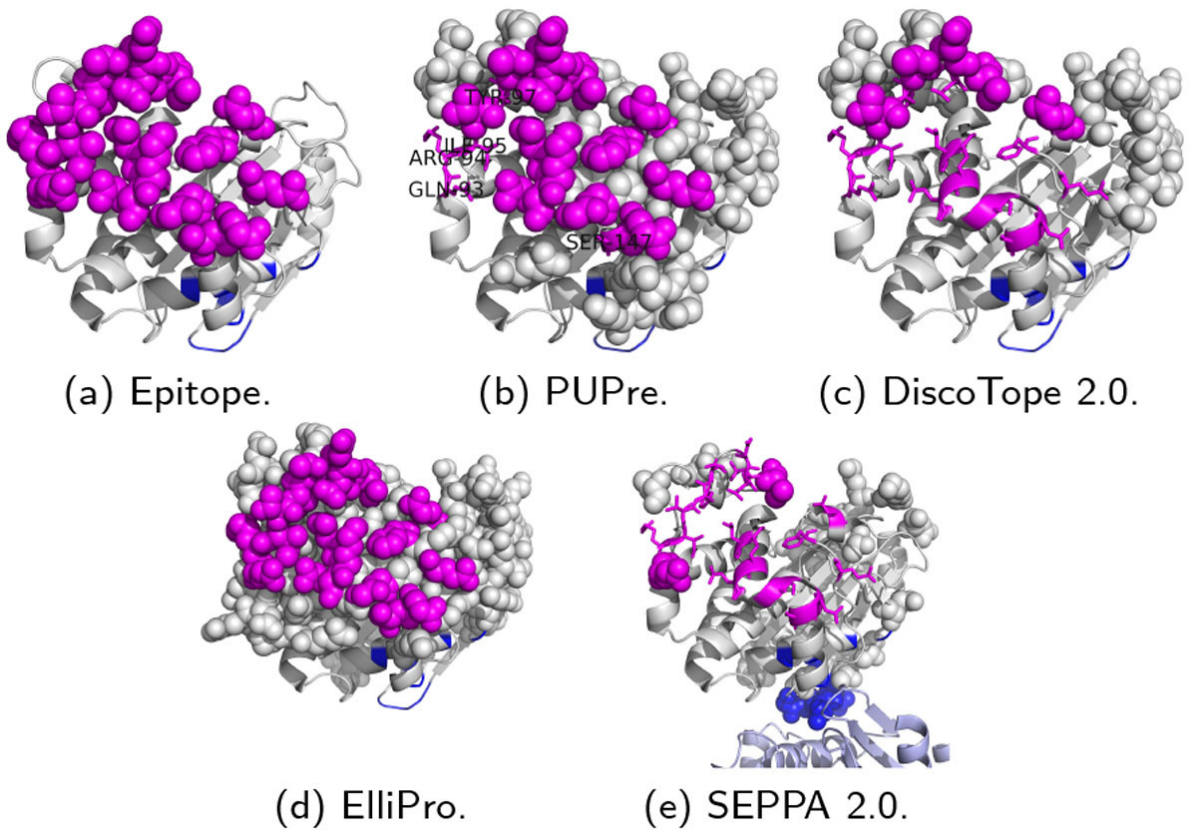
**Epitope prediction on a beta-lactamase unbound structure (PDB ID: 2WK0)**. Magenta denotes the confirmed epitope sites, and blue denotes the internal interactions by other chains; predicted epitopes by each predictor are highlighted by spheres, while the epitope sites that fail to be identified (false negative) are shown as sticks.

ElliPro identified all of the 20 epitope residues, but it also predicted 120 unlabeled residues as epitope residues (Figure 5(d)). PUPre identified 15 out of 20 epitope residues with much higher precision (only 39 false positive residues). Figure 5(e) shows part of Chain B in purple cartoon, and the interaction area is colored blue. Four residues (GLY-52, THR-53, ASN-54, ARG-55) bound by Chain B were wrongly predicted as epitope residues by SEPPA 2.0, while this did not occur with PUPre, DiscoTope 2.0 or ElliPro.

#### Predicted epitopes for Ebola virus antigen

Ebola is a fatal infectious disease that caused a pandemic in Africa in 2014. There are two unbound structures of the matrix proteins of Ebola virus, 1ES6 and 4LD8, stored in PDB. Neither of them can be aligned with any bound structure in PDB with greater than 30% sequence similarity, which means that their bound structures with antibodies have not been determined or published, thus their epitopes cannot be determined from any complex structure data. We attempted to make predictions for the currently unknown epitopes of Ebola antigens through the four structure-based predictors PUPre, DiscoTope 2.0, ElliPro and SEPPA 2.0.

**Figure 6:**
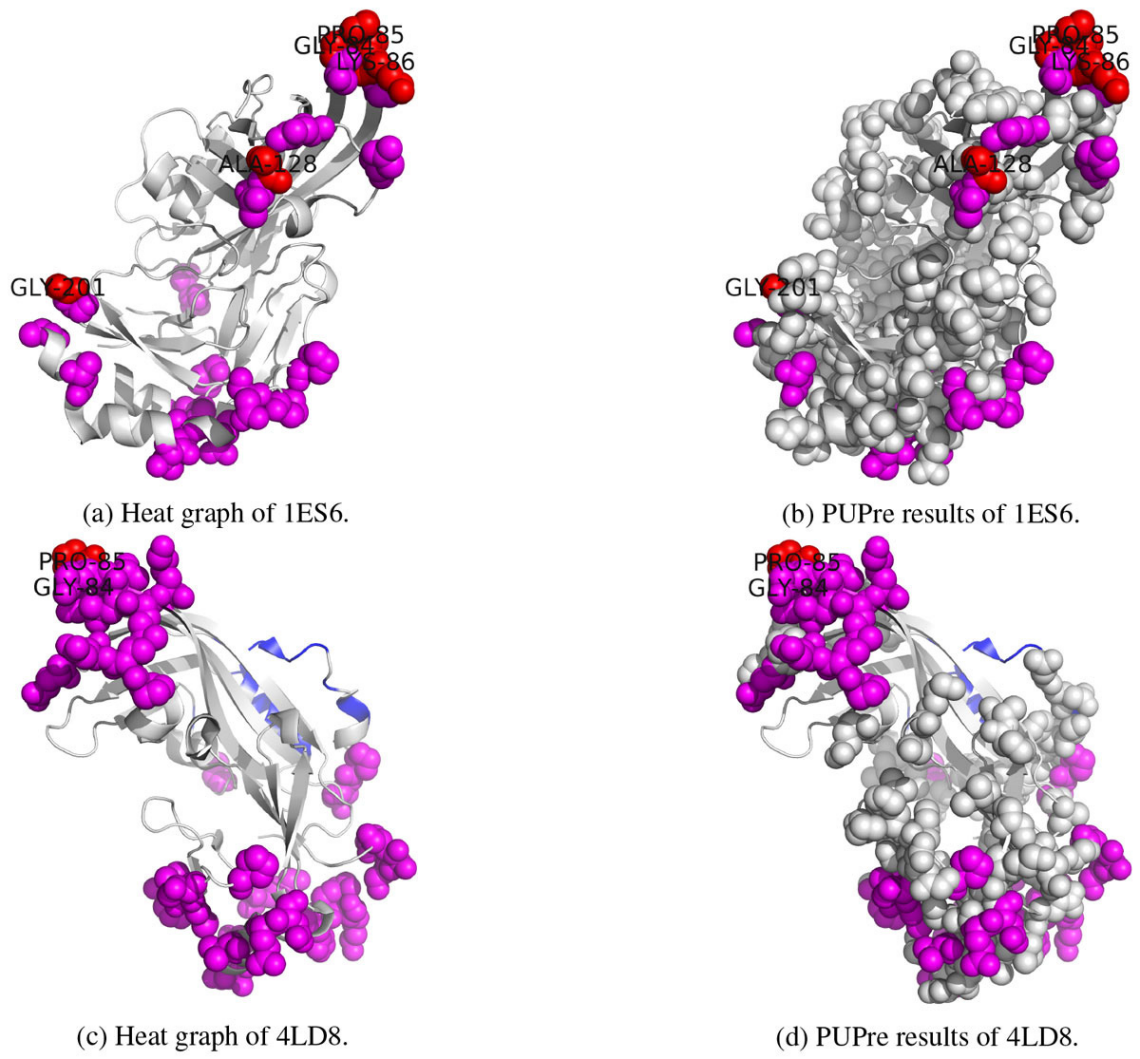
**Epitope prediction on Ebola virus**. Residues which are predicted to be in epitopes by all four methods are in red, those predicted by three methods are in magenta, and hot spot residues are shown in spheres in (a) and (c). Blue represents the internal interactions by other chains. Predicted epitopes by PUPre are highlighted by spheres in (b) and (d).

Each of these methods was first applied to predict epitope residues. Then, the count of a residue predicted as an epitope residue was recorded. It is assumed that if a residue is predicted as an epitope residue by more methods, it is more likely to be a true epitope residue. Hot predictions are highlighted by colored spheres in Figure 6: the red spheres denote those residues predicted by all four prediction methods as epitope sites, and the magenta spheres denote those identified by three predictors as epitope residues. Figure 6(b) and (d) illustrates the prediction results of PUPre. It can be seen that all the residues predicted as epitope residues by three or four predictors (colored in red or magenta) can be identified as epitope sites by PUPre (recall = 100%). ElliPro also identifies all the hot residues, but it recognizes more residues as epitope sites than PUPre: 141 vs 117 for 1ES6, and 126 vs 92 for 4LD8. In addition, ElliPro and DiscoTope 2.0 wrongly predicted some internally interacting residues as epitope residues. This problem does not occur for PUPre.

Taking 1ES6 as an example, GLY-84, PRO-85, LYS-86, ALA-128 and GLY-201 of Chain A were predicted to be epitope residues by all four methods, and some nearby residues, e.g., SER-83, VAL-87, THR-129, GLN-167, GLN-170, ALA-202, ASN-227 and THR-232 were predicted as epitope residues by three methods. It is interesting to see that these residues are spatially close to each other. As aggregated antigenic residues are more likely to constitute an epitope [10], these residues are good candidates for forming novel epitope sites on 1ES6 of the Ebola matrix protein.

## Important features

The identification of important features plays a key role in various areas of biological research [33,34]. Feature analysis is a detailed approach to understanding the particular properties or compound properties of antigen-antibody interfaces that can contrast protein-protein binding sites and the other surface residues. For the purpose of accurately predicting currently unknown epitopes from unbound structures, it is useful for feature analysis to be conducted on a large-scale unbound-state structure data set. Traditional feature analysis has usually been conducted on bound-state structure data sets which introduced bias to the investigation of structural features such as RSA, ASA, PI and B-factor [27]. To understand the unique properties of antigens of different species, we also carried out species-specific feature analyses for virus, bacteria and mammals.

### Top-ranked features

The Wilcoxon rank-sum hypothesis test was used to rank all features extracted from our large-scale unbound structure data set with 18738 residues. The features consist of a total of 239 physico-chemical, evolutionary and structural features. To avoid over-fitting, nine data sets by independent sampling were used.

The top-20 features are summarized in Table 7 (further details can be found in Additional File 4: Table S2). These top-ranked features include structural features, such as ASA, RSA, PI and B-factor (residue-average), evolutionary features (PSSM) and physico-chemical features. Secondary-structure related basic features are not in the list. However, the feature turns and strand (beta sheet) obtained by DSSP are top-ranked 37 (37.44) and 45 (45.11) respectively; the features ARGP820102 and MONM990201, which imply the information extracted from secondary structures, are in the top-20 list.

**Table 7:** Top 20 features selected by Wilcoxon rank-sum hypothesis test.

Feature name	Average rank	Feature name	Average rank
ASA	1.11 ≈ 1	PSSM (LYS)	8.33 ≈ 8
RSA	1.89 ≈ 2	PSSM (ARG)	10.78 ≈ 11
PI	3.00 = 3	JACR890101	12.22 ≈ 12
PSSM (ASN)	5.67 ≈ 6	WARP780101	12.44 ≈ 12
PSSM (ASP)	5.67 ≈ 6	ARGP820102	12.67 ≈ 13
PSSM (GLU)	7.22 ≈ 7	HOPA770101	15.89 ≈ 16
B-factor	7.22 ≈ 7	MONM990201	16.33 ≈ 16
PSSM (GLN)	7.67 ≈ 8	COWR900101	18.44 ≈ 18

JACR890101: Weights from the IFH scale (Jacobs-White, 1989).

WARP780101: Average interactions per side chain atom (Warme-Morgan, 1978).

ARGP820102: Signal sequence helical potential (Argos et al., 1982).

HOPA770101: Hydration number (Hopfinger, 1971).

MONM990201: Averaged turn propensities in a transmembrane helix (Monne et al., 1999).

COWR900101: Hydrophobicity index, 3.0 pH (Cowan-Whittaker, 1990)

The distinction between epitope residues and surface residues in these top ranked features is significant (the p-values are all below 1*e*-9). **ASA and RSA: **the median ASA of epitope residues is 67.7 ^º^*A*^2^, while that of other surface residues is 37.2 ^º^*A*^2^; the median RSA of epitope residues is 43.8% and that of other surface residues is 24.6%. This indicates that epitope residues are more exposed than other surface residues. **PI **is an important feature often taken into account in the identification of epitopes [8,13]. The median PI of epitope residues is 0.709, and that of other surface residues is 0.436, suggesting that epitope is more protrusive than the normal surface. **B-factor **characterizes the mobility of residues, and is claimed to be an effective feature in epitope prediction [9,10]. Normalized B-factor on each antigen was used here, because B-factor may be influenced by the experimental conditions, such as resolution. The median B-factor of epitope sites is 0.31, while that of other surface sites is -0.06, indicating that the epitope sites are more flexible than the surface sites. More details are reported in Additional File 5: Figure S2-S4. Since we assumed that some of the unlabeled residues are undiscovered antigenic residues, the distribution of these features between epitope residues and true non-epitope residues is expected to be more opposed.

**Figure 7:**
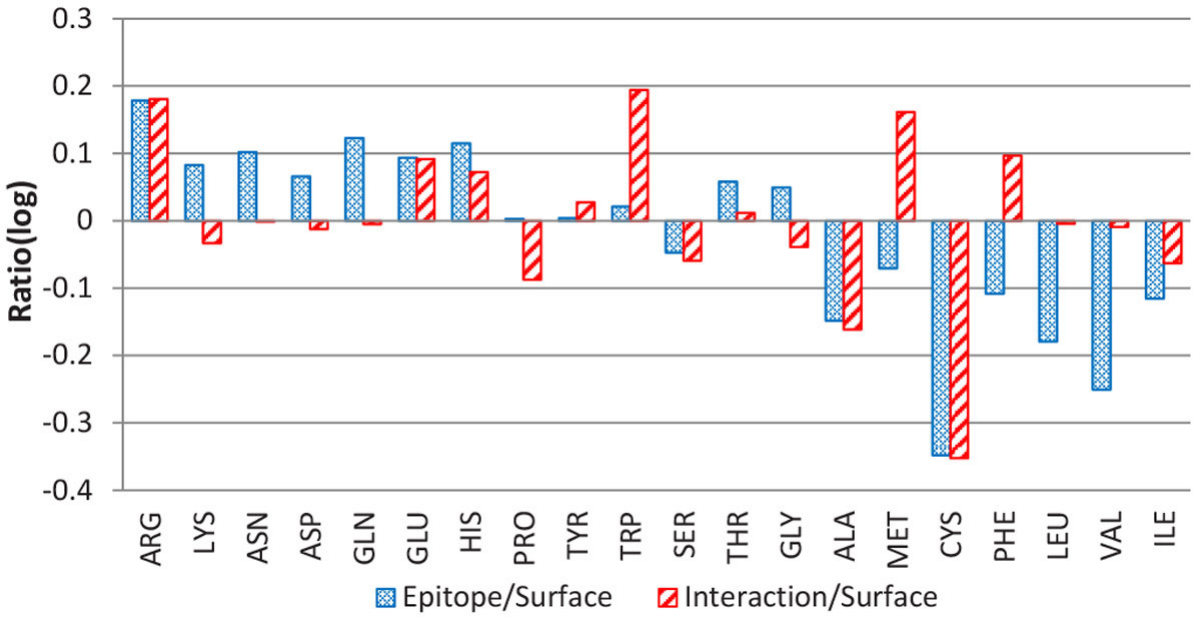
**The ratio of epitope/interface and surface amino acids**. The amino acids are sorted by hydrophobicity: the amino acids on the left side are more hydrophilic, while the amino acids on the right side are more hydrophobic. For the calculation of ratio, refer to Additional File 5: Figure S5.

**Amino acid composition **has long been considered to be an essential feature in identifying epitopes [35,36]. Figure 7 demonstrates the composition of the 20 standard amino acids in epitopes compared with those in internal interactions and other surface areas. It is interesting to see that the most hydrophilic residues (ARG, LYS, ASN, ASP, GLN and GLU) occur significantly more frequently in epitopes than in other surface areas, and the ratios are not less than 1.15. In contrast, all the most hydrophobic residues (ILE, VAL, LEU, PHE, CYS, MET, ALA) clearly exist more frequently in the other surface areas than in the epitope areas with a ratio less than 0.85 (Additional File 5: Figure S5). Most of the other residues in the middle have no significant preference for epitope or normal surface. This reveals that the epitope sites are more hydrophilic than the surface sites (without interfaces within antigens). This observation is also supported by the top-ranked PSSM features in Table 7 where the PSSM of the most hydrophilic residues rank 4th to 10th. As many studies have reported that protein-protein binding interfaces in general cases are dominated by more hydrophobic residues [33,37], it seems that the hydrophilicity preference for amino acids participating in antibody-antigen binding is quite different from the general case.

### Species-specific feature analysis

Species have unique differences in morphology and structure. Investigating whether the epitopes of antigens of different species have distinct characteristics would assist the construction of epitope predictors using species information. We organized the whole data set into seven sub-groups: virus (group 0), parasite (group 1), bacteria (group 2), mammal (group 3), insect (group 4), plant (group 5) and other microbes (group 6). We especially conducted species-specific feature analysis for groups 0, 2 and 3. (The other groups all have a small number of samples, and so were excluded from analysis).

#### Structural features

**Figure 8:**
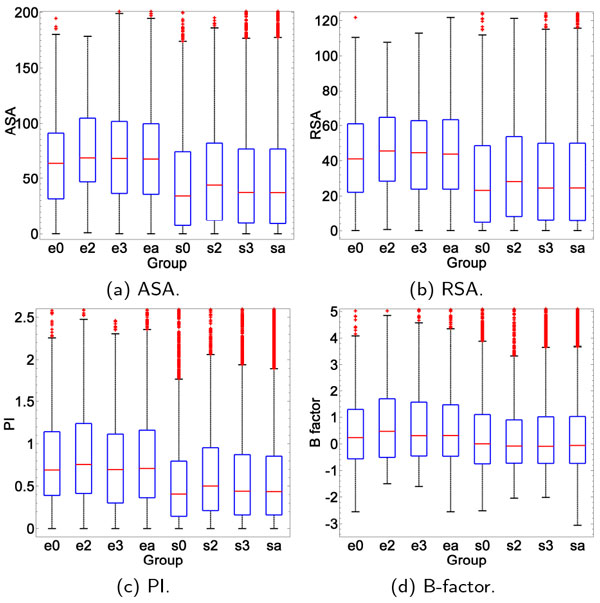
**Species-specific analysis on four structural features**. 0 denotes virus, 2 denotes bacterium, 3 denotes mammals, and 'a' is the overall distribution of all groups. 'e' stands for the epitope group, while 's' represents the surface group.

Figure 8 illustrates the value distribution of structural features ASA, RSA, PI and B-factor on the three species groups. We can see that these features have a similar pattern to that described above: the epitope sites are more exposed, protrusive and flexible. With respect to the feature distribution of epitopes, a difference between species seems to exist but the nature of this difference is not obvious.

Table 8 statistically quantifies the differences. For each feature, the p-values of rank-sum tests for each pair of species--virus (0) vs bacteria (2), virus (0) vs mammal (3), and bacteria (2) vs mammal (3) are presented. The lower the p-value is, the more obvious is the difference. The commonly used threshold of 5% was adopted to tell whether apparent differences exist, those with significant differences are shown in bold. First, we note that the divergence between epitope and surface (p-value less than 1*e - *9) is much larger, suggesting that traditional epitope prediction methods are useful for all species. However, we also find that some pairs of species have significantly small p-values, indicating potential divergence and differences between species. For example, the divergence between virus and the other two groups of species in ASA is significant with p-values of 0.004 and 0.007, but mammals and bacteria seem to have a similar distribution with a p-value of 0.341. PI has a completely contrary trend: the difference between mammals and bacteria is apparent (0.039), while the distribution of both of them resembles that of virus. Thus, integrating species information is likely to be helpful in enhancing prediction performance.

**Table 8:** Difference between virus (e0), bacteria (e2) and mammals (e3) by the rank sum test.

Feature	e0:e2	e0:e3	e2:e3
ASA	**0.004**	**0.007**	0.341
RSA	**0.030**	0.229	0.161
PI	0.320	0.109	**0.039**
B-factor	**0.015**	**0.034**	0.318

Column 'ei:ej' shows the p-value by rank-sum test between groups ei and ej, where i,j can be 0,2 or 3.

#### Amino acid composition

**Figure 9:**
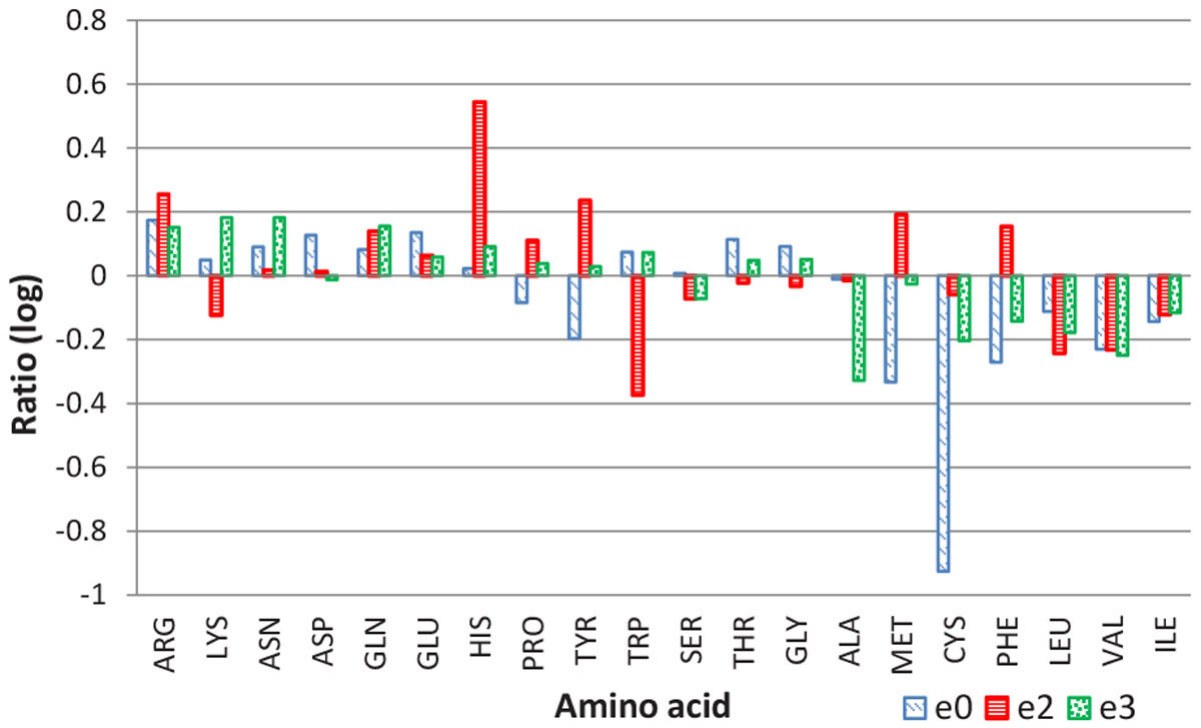
**Species-specific amino acid composition**. e0 is the epitopes of viruses, e2 is the epitopes of bacterium, and e3 is the epitopes of mammals.

We also investigate the amino acid composition of epitopes between different species. It is not surprising that among the three species, hydrophilic residues rather than hydrophobic residues are more likely to constitute epitopes (Figure 9). That is, compared with surface, epitopes are more hydrophilic. The common trend of amino acid composition across species would facilitate a general predictor for all species; however, the ratio of an individual residue suggests the different composition of amino acids in various species. For example, some aromatic residues (e.g.,HIS, TYR and PHE) prefer epitopes of bacteria, while TRP occurs less frequently in the epitopes of bacteria. This phenomenon does not manifest for virus and mammal. In virus, the two aromatic residues--PHE and TYR, and the two sulphur-containing residues (MET and CYS) occur more frequently in surface rather than epitopes. Thus, a species-specific predictor would help improve the prediction performance of epitopes in bacteria and virus for example.

Similarly, the secondary structure distribution of different species exhibits a similar trend, as shown in Additional File 5: Figures S6 and S7, but a specific secondary structure has varies in distribution across species.

## Conclusions

To deal with the issue of incomplete ground truth of training data in B-cell epitope prediction, we have designed a PU learning algorithm based on weighted SVM. A preprocessing procedure was incorporated to remove the internal interactions within the unbound structure of antigens. The integrated framework is named PUPre. A complex-based 10-fold cross-validation process was deployed to evaluate the prediction performance. The results show that PUPre performance exceeds three other commonly used conformational B-cell epitope predictors DiscoTope 2.0, ElliPro and SEPPA 2.0, and two well-designed baseline algorithms, demonstrating the effectiveness of its features, preprocessing procedure and the PU learning algorithm. PUPre was tested on antigens from West Nile virus, dihydrofolate reductase, and beta-lactamase to illustrate the detailed performance of the prediction methods. It was also used for the prediction of unknown epitopes on an antigen of Ebola virus. A species-specific feature analysis was conducted which shows that similar trends exist between epitope and surface in different species, which enables traditional predictors to be useful for all species; the details vary, however, thus refinement by using species information may help to enhance prediction performance. Incomplete training data is a long-neglected but key issue in epitope prediction, as it seriously prevents further performance improvement by traditional methods. PU learning provides a promising direction to pursue to resolve this issue.

## Competing interests

The authors declare that they have no competing interests.

## Authors' contributions

JL proposed the idea of applying PU learning to epitope prediction. JE used his expertise to help construct the species-specific data set. QL provided important suggestions to the design of this PU learning algorithm. JR planned and participated in the entire study from data collection, algorithm design to paper writing. All authors revised and improved the paper, and have read and approved the final manuscript.

## Supplementary Material

Additional File 1 **Data set of unbound structures**. This additional file contains a description of the newly-constructed unbound structure data set. (*.xls) Click here for file

Additional File 2 **Annotated epitope residues on the 92 unbound structures**. This additional file contains the epitope annotations of the unbound structure data set. Zip archive: (*.zip) Click here for file

Additional File 3 **Internally interacting residues of the 92 unbound structures**. This additional file contains the internally interacting residues of the unbound structure data set. Zip archive: (*.zip) Click here for file

Additional File 4 **Supplementary Tables**. This additional file contains Tables S1-S2. (*.pdf) Click here for file

Additional File 5 **Supplementary Figures**. This additional file contains Figures S1-S8. (*.pdf) Click here for file

## Declaration

Publication charges for this article have been funded by ARC Discovery Project DP130102124.

This article has been published as part of *BMC Bioinformatics *Volume 16 Supplement 18, 2015: Joint 26th Genome Informatics Workshop and 14th International Conference on Bioinformatics: Bioinformatics. The full contents of the supplement are available online at http://www.biomedcentral.com/bmcbioinformatics/supplements/16/S18.
