# Supplementary material for: Positive-unlabeled learning for the prediction of conformational B-cell epitopes
Source: BMC Bioinformatics. 2015 Dec 9;16(Suppl 18):S12. doi: 10.1186/1471-2105-16-S18-S12 (PMC4682424; doi:10.1186/1471-2105-16-S18-S12)
Supplement: Additional File 4 — Supplementary Tables. This additional file contains Tables S1-S2. (*.pdf) [file 1471-2105-16-S18-S12-S4.pdf]

ADDITIONAL FILES

Additional File 4 - Supplementary Tables

Jing Ren, Qian Liu, John Ellis and Jinyan Li

Full list of author information is available at the end of the article

Table S1 The performance of structure-based predictors with postprocessing to remove the internally interacting residues from positive predictions. Compared with Table 3, the precision increases for DiscoTope 2.0 and SEPPA 2.0, indicating that some of the internally interacting residues wrongly predicted as epitope sites have been corrected. Also, the slight decrease in recall is because epitopes and internally interacting residues may overlap at their edges (Additional File 5: Figure S8).

Predictor	Recall	Precision	F-score	MCC
DiscoTope2	0.24	0.19	0.21	0.11
ElliPro	0.64	0.12	0.20	0.07
SEPPA2	0.44	0.17	0.24	0.14

Table S2 Details of the top-ranking features.

Feature name	Rank 1	Rank 2	Rank 3	Rank 4	Rank 5	Rank 6	Rank 7	Rank 8	Rank 9	Average rank
ASA	1	1	1	1	1	1	2	1	1	1.11
RSA	2	2	2	2	2	2	1	2	2	1.89
PI	3	3	3	3	3	3	3	3	3	3.00
PSSM (Asn)	4	5	6	4	7	6	5	7	7	5.67
PSSM (Asp)	5	4	5	10	5	4	7	5	6	5.67
PSSM (Glu)	8	6	10	8	6	5	8	9	5	7.22
B factor	6	13	4	5	4	21	4	4	4	7.22
PSSM (Gln)	7	7	7	9	8	7	6	10	8	7.67
PSSM (Lys)	9	9	9	6	9	9	9	6	9	8.33
PSSM(Arg)	11	12	14	7	10	10	15	8	10	10.78
JACR890101	15	10	12	13	12	11	11	13	13	12.22
WARP780101	16	8	8	12	16	14	13	14	11	12.44
ARGP820102	17	11	11	15	11	12	14	11	12	12.67
HOPA770101	20	14	13	22	13	8	23	15	15	15.89
MONM990201	21	15	15	11	15	19	18	16	17	16.33
COWR900101	22	17	22	17	17	15	16	20	20	18.44
