# Supplementary material for: Positive-unlabeled learning for the prediction of conformational B-cell epitopes
Source: BMC Bioinformatics. 2015 Dec 9;16(Suppl 18):S12. doi: 10.1186/1471-2105-16-S18-S12 (PMC4682424; doi:10.1186/1471-2105-16-S18-S12)
Supplement: Additional File 5 — Supplementary Figures. This additional file contains Figures S1-S8. (*.pdf) [file 1471-2105-16-S18-S12-S5.pdf]

ADDITIONAL FILES

Additional File 5 - Supplementary Figures

Jing Ren, Qian Liu, John Ellis and Jinyan Li

Full list of author information is available at the end of the article

Figure S1: The distribution of residues on RSA and PI.

Figure S1 presents the distribution of positive and unlabeled points on RSA and PI.

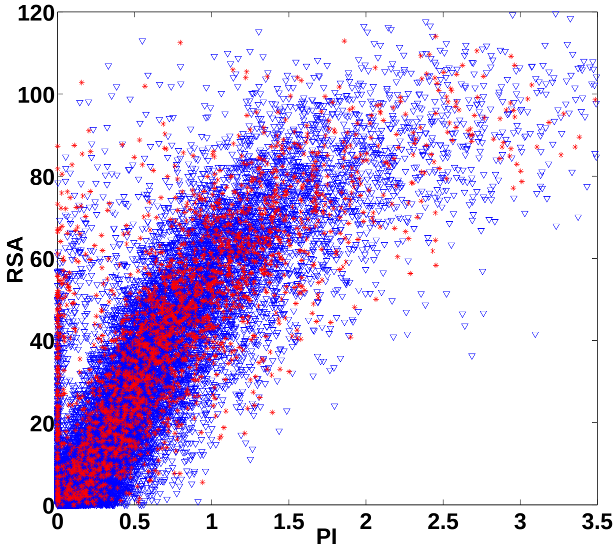

Figure S1 The distribution of residues on RSA and PI. The confirmed epitope residues are colored in red, while the unlabeled residues are colored in blue.

Figure S2: The distribution of ASA and RSA.

In most cases, a residue needs to be exposed to be identified by antibodies [1]. Figure S2 illustrates the distribution of ASA and RSA in epitope and other surface residues (ASA>0). The median ASA of epitope residues is 67.7 $\mathring{A}^2$, while that of other surface residues is 37.2 $\mathring{A}^2$; the median RSA of epitope residues is 43.8%, but that of other surface residues is 24.6%. The differences in these two features between epitope residues and surface residues are significant with p-values (rank-sum test) of 3.1e-127 and 1.3e-124 respectively. This indicates that epitope residues are more exposed than other surface residues.

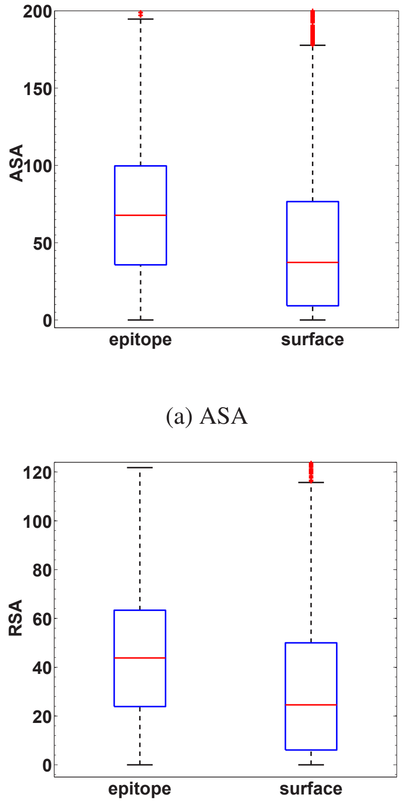

(a) ASA

(b) RSA

Figure S2 ASA and RSA distribution.

Figure S3: The distribution of PI.

PI ranks 3rd in Additional File 4: Table S2. Previous studies also suggested that this feature is important for the identification and prediction of epitopes [2, 3]. The distribution of PI is shown in Figure S3: the median PI of epitope residues is 0.709, and that of other surface residues is 0.436; their difference is obvious with a p-value of 1.2e-74. It suggests that epitope is more protrusive than surface.

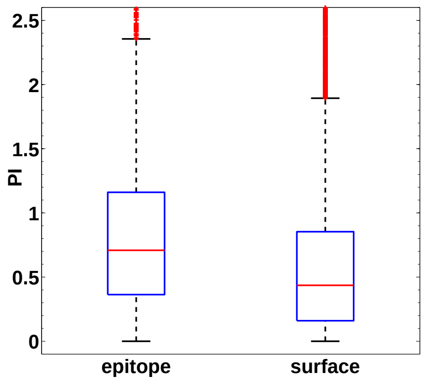

Figure S3 PI distribution.

Figure S4: The distribution of B factor.

B factor characterizes the mobility of residues, and is claimed to be an effective feature in epitope prediction [4, 5]. It ranks 7th in Additional File 4: Table S2. Figure S4 demonstrates the distribution of the normalized B factor in epitope and surface residues. Normalized B factor on each antigen is used here, because B factor may be influenced by the determination conditions, such as resolution. The median B factor of epitope sites is 0.31, while that of other surface sites is -0.06. Their distribution is remarkably different as the p-value between them is 4.0e-30, which implies that the epitope sites are more flexible than the surface sites.

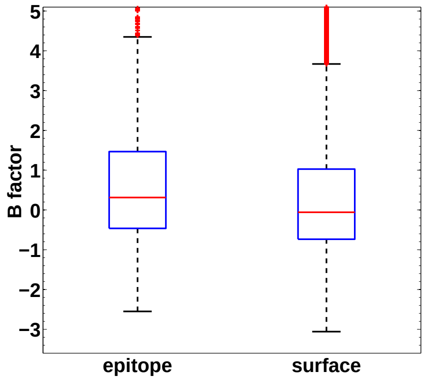

Figure S4 B factor distribution.

Figure S5: Amino acid composition

Figure S5 shows the ratio of amino acids between the epitope (or interaction) and surface residues.

The ratios are calculated as:

$$EpitopeSurfaceRatio\,(Ri) = \frac{Ri_{epitope}/\sum Ri_{epitope}}{Ri_{surface}/\sum Ri_{surface}},\ \text{and:}$$

$$InteractionSurfaceRatio\,(Ri) = \frac{Ri_{interaction}/\sum Ri_{interaction}}{Ri_{surface}/\sum Ri_{surface}}$$

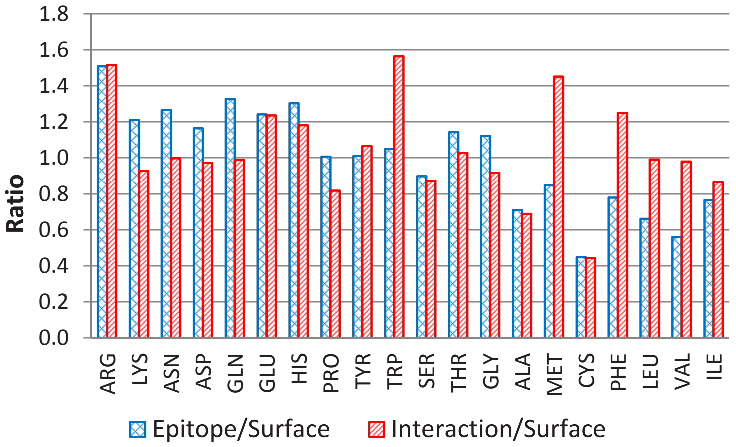

Figure S5 The ratio between the epitope (or interaction) and surface amino acids. The amino acids are sorted by hydrophobicity: the amino acids on the left side are more hydrophilic, while the amino acids on the right side are more hydrophobic.

Figure S6: Secondary structure

Secondary structure is another conventional feature used in epitope prediction [6]. Figure S6 shows the distribution of secondary structures. Compared with surface sites, the epitope sites are richer in turn and shorter in beta sheets. Additionally, epitope and interaction demonstrate a obviously different preference, for example, interactions contain more alpha helix than epitopes.

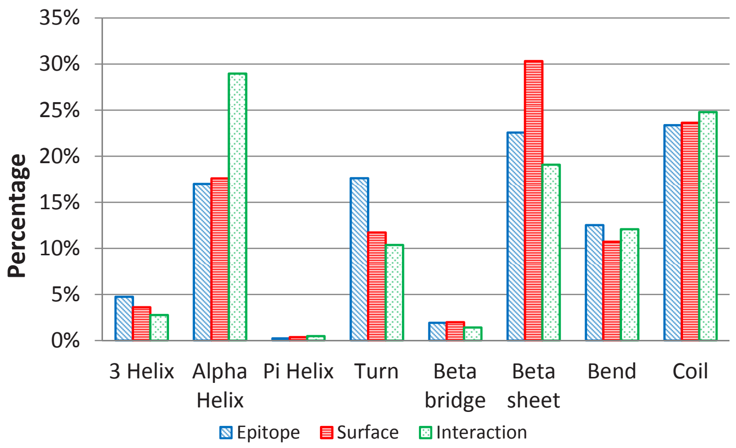

Figure S6 The distribution of secondary structures in epitope, surface and internal interaction.

Figure S7: Secondary structure composition in different species

Figure S7 presents the distribution of secondary structures in different species.

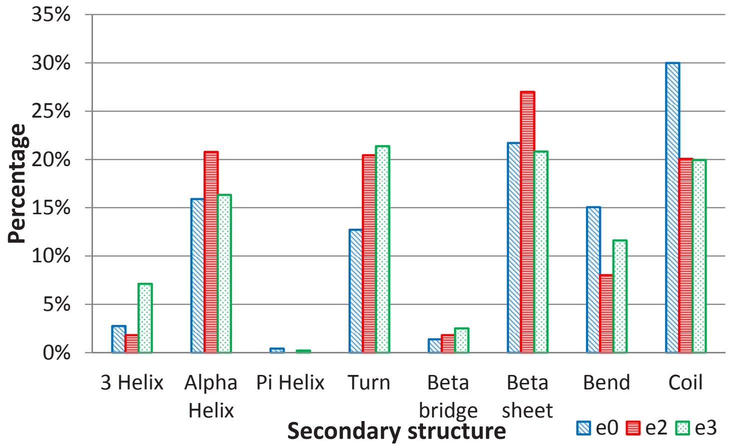

Figure S7 The distribution of secondary structures in different species. e0 denotes virus, e2 denotes bacterium and e3 denotes mammals.

Figure S8: Overlapping between epitopes and internal interactions.

Epitopes and internally interacting residues may overlap in their edges as shown in Figure S8. This is the reason for the slight decrease in recall in Additional File 4: Table S1. Of all the 1648 internally interacting residues in our unbound data set, 165 overlap the epitope residues.

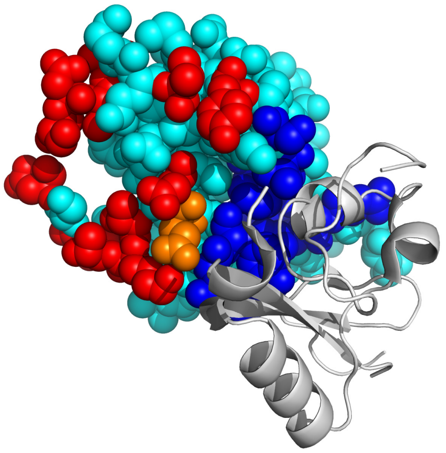

Figure S8 Overlapping between epitopes and internal interactions in 3K7B. The epitope is colored in red, the internally interacting residues are colored blue, and the overlapping residues are colored orange. The other chain that interacts with the target chain is shown in gray.

Author details

References

1. Kulkarni-Kale, U., Bhosle, S., Kolaskar, A.S.: CEP: A conformational epitope prediction server. Nucleic Acids Research **33**(Suppl 2), 168–171 (2005)
2. Thornton, J.M., Edwards, M.S., Taylor, W.R., Barlow, D.J.: Location of 'continuous' antigenic determinants in the protruding regions of proteins. The EMBO Journal **5**(2), 409 (1986)

3. Ponomarenko, J., Bui, H.-H., Li, W., Fusseder, N., Bourne, P.E., Sette, A., Peters, B.: ElliPro: A new structure-based tool for the prediction of antibody epitopes. BMC Bioinformatics **9**(1), 514 (2008)
4. Liu, R., Hu, J.: Prediction of discontinuous B-cell epitopes using logistic regression and structural information. J Proteomics Bioinform **4**, 010–015 (2011)
5. Ren, J., Liu, Q., Ellis, J., Li, J.: Tertiary structure-based prediction of conformational B-cell epitopes through B factors. Bioinformatics **30**(12), 264–273 (2014)
6. Pellequer, J.-L., Westhof, E., Van Regenmortel, M.H.V.: Correlation between the location of antigenic sites and the prediction of turns in proteins. Immunology Letters **36**(1), 83–99 (1993)
